# Prognostic evaluation of esophageal cancer patients with stages I-III

**DOI:** 10.18632/aging.103532

**Published:** 2020-07-23

**Authors:** Meng-jun Qiu, Sheng-li Yang, Meng-meng Wang, Ya-nan Li, Xin Jiang, Zao-zao Huang, Zhi-fan Xiong

**Affiliations:** 1 Division of Gastroenterology, Liyuan Hospital, Tongji Medical College, Huazhong University of Science and Technology, Wuhan 430077, China; 2 Cancer Center, Union Hospital, Tongji Medical College, Huazhong University of Science and Technology, Wuhan 430022, China; 3 Yangchunhu Community Hospital, Liyuan Hospital, Tongji Medical College, Huazhong University of Science and Technology, Wuhan 430077, China

**Keywords:** esophageal cancer, SEER database, surgery, nomogram, prognosis

## Abstract

Purpose: The purpose of this study was to investigate the impact of clinicopathological factors and treatments on the overall survival (OS) and esophageal cancer-specific survival (ECSS) of stages I-III esophageal cancer (EC) patients and to establish a prognostic visual nomogram. Methods: We collected clinical data of patients diagnosed with stages I-III EC without receiving chemotherapy from 2004 to 2014 from the Surveillance, Epidemiology, and End Results (SEER) database. Prognoses were analyzed using the R language software, and nomograms were obtained according to the visual processing logistic regression model, which was verified using the Harrell C-index, receiver operating characteristic (ROC) curve, and calibration curve. Results: A total of 4,305 patients were selected, mostly white males. Most patients were over 60 years old and old age predicted poor prognosis. EC, primarily adenocarcinoma, occurred mostly in the lower third of the esophagus. About half of the patients had T1 (58.00%) and grade II (50.41%) cancer. Of all the patients, 2,448 was treated with surgery and the majority (n = 1,476; 64.85%) of these patients had stage I EC. Stages I-III patients underwent surgery had significantly better OS and ECSS, and endoscopic therapy was associated with the best outcome amongst all the surgical methods. 3.67% of the patients received radiotherapy, predominantly postoperative radiotherapy (2.69%). Older age, squamous cell carcinoma, overlapping lesion of the esophagus, and grades II and III were high-risk factors for poor OS and ECSS for stage I patients, whereas endoscopic therapy, esophagectomy, and esophagectomy with gastrectomy were low-risk factors. Stage II patients with older age, male sex, T3, N1, and grades II and III had shorter OS and ECSS, but patients with any surgical treatment had significantly longer OS and ECSS. T4, N1, and grade III correlated negatively with OS and ECSS in stage III patients, and any surgical treatment correlated positively with longer OS and ECSS. The OS and ECSS rates of stages I-III EC patients with a total score of more than 150 points in the nomogram were both only 40% after 3 years and 30% after 5 years. The C-index, ROC curve, and calibration curve indicated that the nomograms established in this study were suitable to assess patient prognosis. Conclusion: The nomogram established in this study is an effective clinical tool to predict the prognosis of stages I-III EC patients without chemotherapy.

## INTRODUCTION

In the 2018 global cancer statistics, esophageal cancer (EC) is the seventh most widespread cancer in the world and the sixth leading cause of cancer deaths [1]. The most common histological subtypes of EC are squamous cell carcinoma (SCC) and adenocarcinoma (AC). SCC accounts for 90% of EC, but the incidence of AC is increasing in Western countries [2, 3]. Smoking, drinking, and hot drinks are the primary risk factors for SCC, whereas gastroesophageal reflux disease and obesity are the major risk factors for AC [4, 5]. In the past ten years, with the advancement of diagnostic and treatment technologies, both the incidence and mortality of EC have been decreasing. For stages I-III EC patients, based on the patient’s tumor status and specific stage, either radical esophageal resection or radical surgery is performed before or after the adjuvant chemoradiotherapy. Due to the insidious onset, highly invasive properties, and rapid progress, the prognosis of EC patients remains poor, with 5-year survival rates ranging from 20% to 30% [6, 7]. For advanced EC patients with distant metastasis, improvement of the quality of life after treatment remains the priority. Therefore, it is important to study the heterogeneity of stages I-III EC and the differences in prognosis among individual patients after receiving different surgical treatments.

A nomogram is developed based on a multivariate regression model. Using scaled line segments, various forecast indicators are listed and scored, and the total score of all indicators can be used to predict the outcome [8]. In general, the Harrell C-index and the area under the curve (AUC) of the receiver operating characteristic (ROC) curve are used to indicate the predictive power of the nomogram. Model validation and calibration curve are also important.

This study used clinical data from stages I-III EC patients who did not receive chemotherapy. The data were gathered from the Surveillance, Epidemiology, and End Results (SEER) database and used for analysis of clinical parameters affecting the overall survival (OS) and EC-specific survival (ECSS) of EC patients. In addition, a dynamic prediction model for EC was constructed and validated.

## RESULTS

### Clinical pathological characteristics

**Figure 1:**
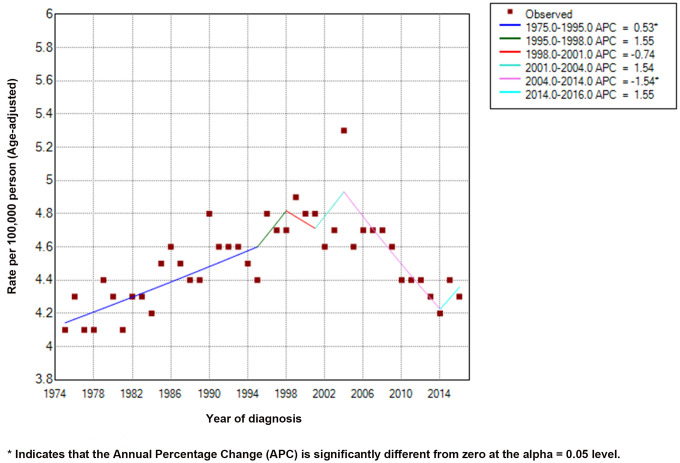
**Incidence rates (Age-adjusted) and annual percentage change trends of esophageal cancer.**

**Figure 2:**
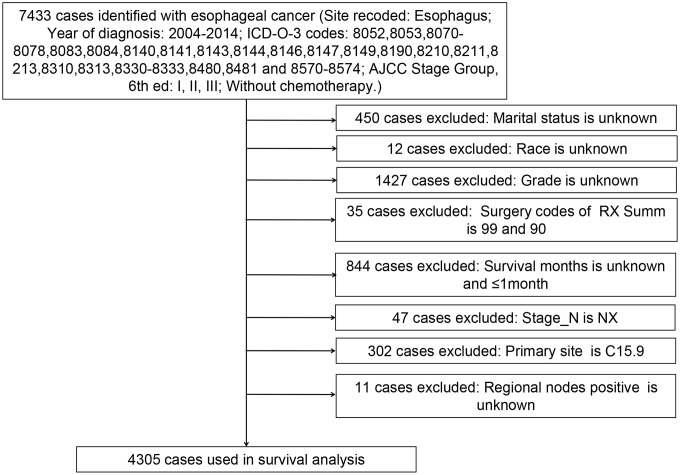
**Flow diagram of esophageal cancer patient selection from the SEER database between 2004 and 2014.**

We calculated the APCs of EC between 1975 and 2016 (Figure 1). The incidence of EC decreased significantly from 2004 to 2014 (APC = -1.54) and therefore, we analyzed the prognosis of EC patients during this decade. With the strict inclusion and exclusion criteria, 4,305 of 7,433 patients were selected for our analysis (Figure 2). The demographic and clinical characteristics of these patients are shown in Table 1. Most of the selected patients were over 50 years old (96.17%), mainly between the ages of 60 to 79. Male patients were about three times as many as female patients, and white patients accounted for the majority (85.41%). The most common site of EC was in the lower third of the esophagus (65.66%). The majority of cases were AC (64.29%). At the time of diagnosis, there were 2,276 stage I patients (52.87%), 1,182 stage II patients (27.46%), and 847 stage III patients (19.67%). Most patients had stage T1 (58.00%) and grade II (50.41%). 77.26% of patients have no basic diseases. Of the total of 4,305 patients, none had distant metastases, and only 24.65% had lymph node metastases. 56.86% of patients underwent surgery, of which 27.40% underwent esophagectomy with gastrectomy. Some patients received endoscopic therapy (10.64%), which consisted of destruction, resection, or the combination of destruction and resection under various endoscopes, such as photodynamic therapy, cryosurgery, laser excision, laser ablation, and electrocautery. Other patients underwent esophagectomy (17.70%), esophagectomy with laryngectomy (0.91%), or the combination of both surgeries (0.21%). Only 3.67% of patients received different types of radiotherapy, largely postoperative radiotherapy (2.69%).

**Table 1:** Characteristics of esophageal cancer patients with stages I-III (n=4,305).

**Characteristics**	**No. of patient (%)**	**No surgery (n=1857) No. (%)**	**Surgery (n=2448) No. (%)**	***P* value**
Age (years)				<0.001
<50	165 (3.83)	52 (31.52)	113 (68.48)	
50-59	649 (15.08)	200 (30.82)	449 (69.18)	
60-69	1188 (27.60)	378 (31.82)	810 (68.18)	
70-79	1256 (29.17)	489 (38.93)	767 (61.07)	
80≤	1047 (24.32)	738 (70.49)	309 (29.51)	
Sex				<0.001
Female	1068 (24.81)	586 (54.87)	482 (45.13)	
Male	3237 (75.19)	1271 (39.26)	1966 (60.74)	
Race				<0.001
White	3677 (85.41)	1472 (40.03)	2205 (59.97)	
Black	416 (9.66)	279 (67.07)	137 (32.93)	
Other	212 (4.93)	106 (50%)	106 (50%)	
Marital status				<0.001
Married	2437 (56.61)	835 (34.26)	1602 (65.74)	
Unmarried	1868 (43.39)	1022 (54.71)	846 (45.29)	
ICD-O-3 Hist/Behav, malignant				<0.001
Squamous cell carcinoma	1537 (35.71)	920 (59.86)	617 (40.14)	
Adenocarcinoma	2768 (64.29)	937 (33.85)	1831 (66.15)	
Primary Site				<0.001
Upper third of the esophagus	358 (8.32)	243 (67.88)	115 (32.12)	
Middle third of the esophagus	999 (23.21)	547 (54.75)	452 (45.25)	
Lower third of the esophagus	2827 (65.66)	1003 (35.48)	1824 (64.52)	
Overlapping lesion of the esophagus	121 (2.81)	64 (52.89)	57 (47.11)	
RX Summ—Surg Prim Site				
No surgery	1857 (43.14)	-	-	
Endoscopic therapy	458 (10.64)	-	-	
Esophagectomy	762 (17.70)	-	-	
With gastrectomy	1180 (27.40)	-	-	
With laryngectomy	39 (0.91)	-	-	
Combination	9 (0.21)	-	-	
Radiation sequence with surgery				<0.001
No radiation	4147 (96.33)	1841 (44.39)	2306 (55.61)	
Radiation after surgery	116 (2.69)	14 (12.07)	102 (87.93)	
Radiation prior to surgery	37 (0.86)	2 (5.41)	35 (94.59)	
Radiation before and after surgery	5 (0.12)	0 (0%)	5 (100%)	
AJCC Staging Group, 6^th^ ed				
				<0.001
I	2276 (52.87)	800 (35.15)	1476 (64.85)	
II	1182 (27.46)	538 (45.52)	644 (54.48)	
III	847 (19.67)	519 (61.28)	328 (38.72)	
Stage_T				<010.0
T1	2497 (58.00)	931 (37.28)	1566 (62.72)	
T2	525 (12.20)	212 (40.38)	313 (59.62)	
T3	960 (22.30)	458 (47.71)	502 (52.29)	
T4	323 (7.50)	256 (79.26)	67 (20.74)	
Stage_N				<0.001
N0	3244 (75.35)	1272 (39.21)	1972 (60.79)	
N1	1061 (24.65)	585 (55.14)	476 (44.86)	
Stage_M				-
M0	4305 (100%)	1857 (43.14)	2448 (56.86)	
Grade				<0.001
Well differentiated, Grade I	516 (11.99)	135 (26.16)	381 (73.84)	
Moderately differentiated, Grade II	2170 (50.41)	924 (42.58)	1246 (57.42)	
Poorly differentiated, Grade III	1569 (36.44)	782 (49.84)	787 (50.16)	
Undifferentiated, anaplastic, Grade IV	50 (1.16)	16 (32)	34 (68)	
Having basic diseases				0.96
No	3326 (77.26)	1434 (43.11)	1892 (56.89)	
Yes	979 (22.74)	423 (43.21)	556 (56.79)	

To exclude bias from baseline characteristics of the subjects due to our strict inclusion criteria, the demographic and clinical characteristics of the 7,743 patients were also calculated by age, sex, pathological type, and clinical staging (Supplementary Table 1). Consistent with the results from the selected patients for analysis (Table 1), the total EC patients were mainly over 60 years old (81.44%), male (74.85%), AC (62.72%), and at stage I (56.05%).

The baseline characteristics of patients in the surgery and no-surgery groups are summarized in Table 1. Of the 2,448 patients treated with surgery, 1,476 (60.29%) had stage I EC, 644 (26.31%) had stage II EC, and 328 (13.39%) had stage III EC. Of the 1,857 patients without surgery, 800 (43.08%) had stage I EC, 538 (28.97%) had stage II EC, and 519 (27.95%) had stage III EC. Proportion of patients treated with surgery varies by clinical staging (*P* < 0.001), and patients with stages I and II had larger proportion of surgery. There was no correlation between patients with basic diseases and surgery (*P* = 0.96).

### Patient prognosis analysis

**Figure 3:**
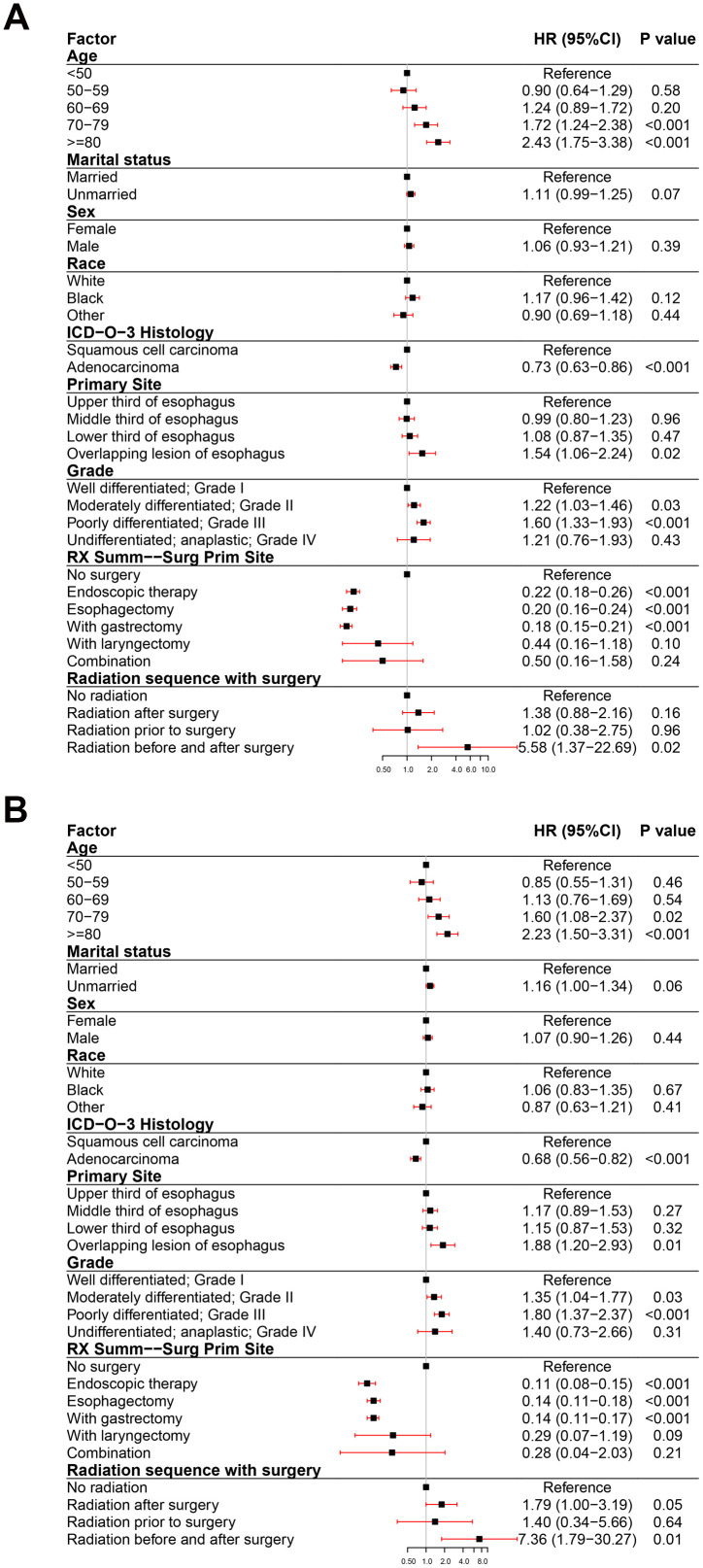
**Forest plot of the multivariate analysis data of stage I esophageal cancer patients using the Cox proportional hazards model.** (**A**) Multivariate analysis of the overall survival for stage I EC patients. (**B**) Multivariate analysis of the esophageal cancer-specific survival for stage I EC patients.

**Figure 4:**
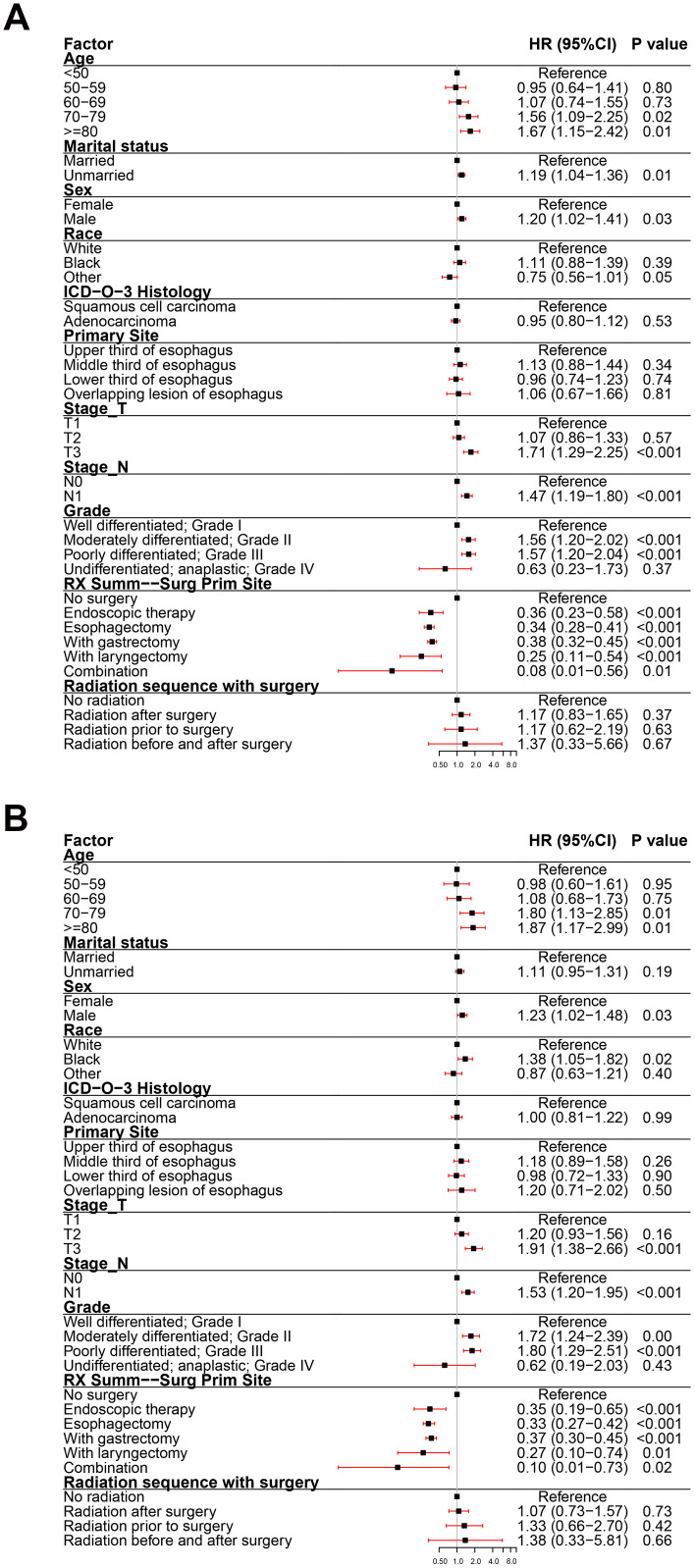
**Forest plot of the multivariate analysis data of stage II esophageal cancer patients using the Cox proportional hazards model.** (**A**) Multivariate analysis of the overall survival for stage II EC patients. (**B**) Multivariate analysis of the esophageal cancer-specific survival for stage II EC patients.

**Figure 5:**
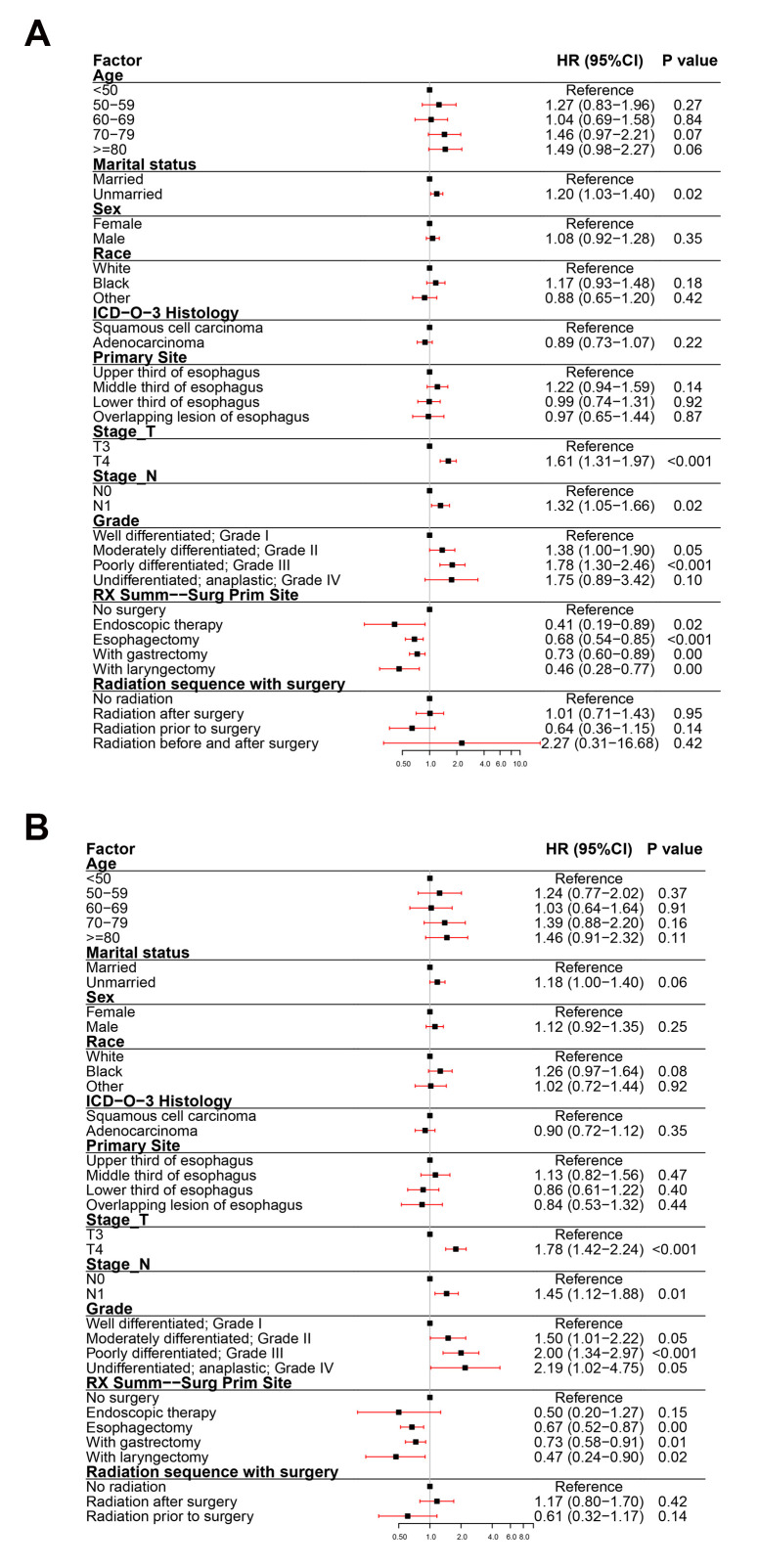
**Forest plot of the multivariate analysis data of stage III esophageal cancer patients using the Cox proportional hazards model.** (**A**) Multivariate analysis of the overall survival for stage III EC patients. (**B**) Multivariate analysis of the esophageal cancer-specific survival for stage III EC patients.

To assess the impact of all variables on the prognosis of EC, we performed multivariate analyses of OS and ECSS in patients at different clinical stage. The multivariate analyses of OS (Figure 3A) and ECSS (Figure 3B) of stage I patients showed that poor prognosis was associated with age (patients aged 70-79 years and over 80 years compared to patients younger than 50 years), pathological type (SCC compared to AC), primary site (overlapping lesion of the esophagus compared to the upper third of the esophagus), grade (grades II and III compared to grade I), and radiotherapy (radiation before and after surgery compared to no radiation). Good prognosis was associated with surgery (endoscopic therapy, esophagectomy, and esophagectomy with gastrectomy compared to no-surgery). The multivariate analysis of OS (Figure 4A) of patients with stage II EC revealed that poor prognosis was related to age (patients aged 70-79 years and over 80 years compared to patients younger than 50 years), marital status (unmarried compared to married), sex (male compared to female), T_stage (T3 compared to T1), N_stage (N1 compared to N0), and grade (grades II and III compared to grade I). Surgery (surgery compared to no-surgery) was associated with good prognosis. Unlike the OS results, the multivariate analysis of the ECSS (Figure 4B) of stage II patients showed that marital status had no impact on prognosis and race (black compared to white) was related to poor prognosis. The multivariate analysis of OS (Figure 5A) of stage III patients indicated that poor prognosis was related to marital status (unmarried compared to married), T_stage (T4 compared to T3), N_stage (N1 compared to N0), and grade (grade III compared to grade I). Surgery (surgery compared to no-surgery) was associated with good prognosis. On the contrary, multivariate analysis of ECSS (Figure 5B) of stage III patients showed that marital status had no impact on prognosis.

**Figure 6:**
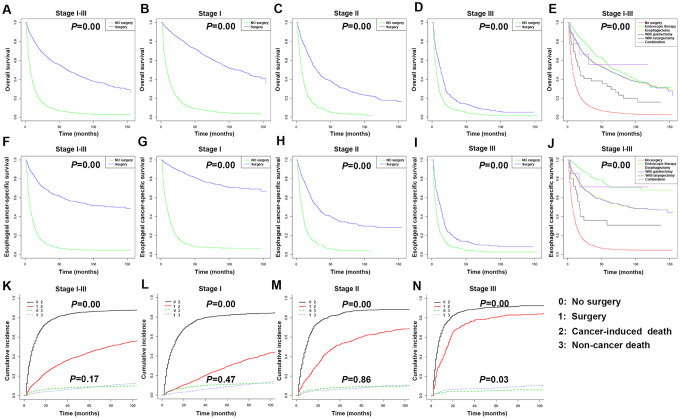
**Kaplan-Meier survival analyses for overall survival (OS) and esophageal cancer-specific survival (ECSS) in stages I-III esophageal cancer (EC) patients underwent different types of surgery.** (**A**) Survival curve for OS in stages I-III EC patients. (**B**) Survival curve for OS in stage I EC patients. (**C**) Survival curve for OS in stage II EC patients. (**D**) Survival curve for OS in stage III EC patients. (**E**) Survival curve for OS based on surgery type in stages I-III EC patients. (**F**) Survival curve for ECSS in stages I-III EC patients. (**G**) Survival curve for ECSS in stage I EC patients. (**H**) Survival curve for ECSS in stage II EC patients. (**I**) Survival curve for ECSS in stage III EC patients. (**J**) Survival curve for ECSS based on surgery type in stages I-III EC patients. (**K**) Competitive risk of cancer-induced deaths and non-cancer-related deaths of stages I-III EC patients. (**L**) Competitive risk of cancer-induced deaths and non-cancer-related deaths of stage I EC patients. (**M**) Competitive risk of cancer-induced deaths and non-cancer-related deaths of stage II EC patients. (**N**) Competitive risk of cancer-induced deaths and non-cancer-related deaths of stage III EC patients.

The Kaplan-Meier survival curve revealed that there were significant differences in OS and ECSS between the surgical group and no-surgery groups of EC patients with stages I-III and each stage (*P* < 0.05) (Figure 6A–6D, 6F–6I). The 3- and 5-year OS rates of stages I-III patients without surgery were 10.30% (95% CI: 8.99-11.80) and 5.53% (95% CI: 4.53-6.75), respectively. The 3- and 5-year OS rates of stages I-III patients with surgery were 60.60% (95% CI: 58.60-62.60) and 50.50% (95% CI: 48.50-52.60), respectively. The 3- and 5-year ECSS rates of stages I-III patients without surgery were 9.80% (95% CI: 8.36-11.50) and 5.71% (95% CI: 4.56-7.16), respectively. The 3- and 5-year ECSS rates of stages I-III patients with surgery were 65.40% (95% CI: 63.30-67.60) and 58.40% (95% CI: 56.10-60.70), respectively. To study the impact of surgical therapy, each of the five surgical methods used in EC were compared to the no-surgery group. Per the Kaplan-Meier survival curve, both OS and ECSS of patients who underwent endoscopic therapy were significantly longer compared to patients received other types of surgery or without surgery (*P* < 0.05). The OS and ECSS were similar between patients underwent esophagectomy and patients received esophagectomy with gastrectomy (Figure 6E, 6J). The hazard ratios (HR) in the esophagectomy group versus the endoscopic therapy group were 1.25 (OS) and 2.13 (ECSS) (*P* < 0.001). The HR in the esophagectomy with gastrectomy group versus the endoscopic therapy group were 1.26 (OS) and 2.13 (ECSS) (*P* < 0.001). The HR in the esophagectomy with laryngectomy group versus the endoscopic therapy group were 2.28 (OS) and 4.15 (ECSS) (*P* < 0.001).

We further compared the prognosis of patients at different clinical stage after endoscopic therapy, esophagectomy, or esophagectomy with gastrectomy (Supplementary Figure 1). Per the Kaplan-Meier survival curve, the OS and ECSS of stages I-III patients who underwent endoscopic therapy were significantly longer than those subjected to esophagectomy or esophagectomy with gastrectomy. The HR in the esophagectomy group versus the endoscopic therapy group were 1.13 (OS) and 1.59 (ECSS) (*P* < 0.05). The HR in the esophagectomy with gastrectomy group versus the endoscopic therapy group were 1.15 (OS) and 1.67 (ECSS) (*P* < 0.05). As the stage I patients underwent endoscopic therapy had a poor OS, the HR in the esophagectomy group versus the endoscopic therapy group was 0.87 (OS) (*P* < 0.05). The HR in the esophagectomy with gastrectomy group versus the endoscopic therapy group was 0.73 (OS) (*P* < 0.05). There were no significant differences in OS and ECSS among the three surgical methods in stages II and III patients. The patient deaths were divided into cancer-induced deaths and non-cancer-related deaths, such as heart disease and cerebrovascular disease, to analyze the impact of the different treatment methods on specific OS. According to the Kaplan-Meier survival curve, each stage of EC patients, as well as the total stages I-III patients, in the surgical groups had significantly reduced risks of cancer-induced deaths compared to patients at the same clinical stage but without surgery (*P* < 0.05). Stages I and II patients, as well as the overall stages I-III patients, in the surgery group had no change in the risk of non-cancer related deaths compared to patients at the same clinical stage but without surgery (*P* > 0.05); however, stage III patients in the surgery group had a higher risk of non-cancer related deaths compared to stage III patients without surgery (Figure 6K–6N).

**Figure 7:**
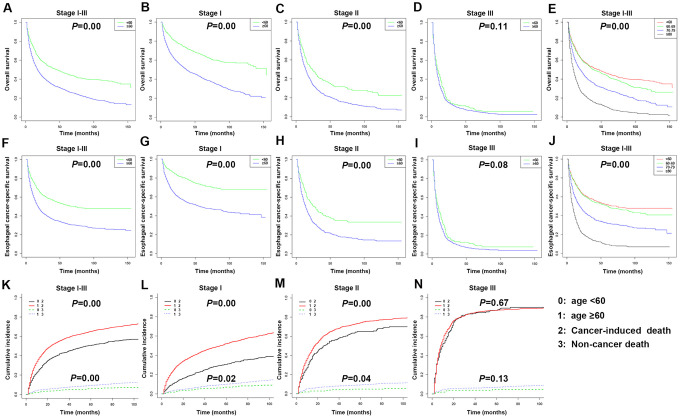
**Kaplan-Meier survival analyses for overall survival (OS) and esophageal cancer-specific survival (ECSS) in stages I-III patients with esophageal cancer (EC) based on age.** (**A**) Survival curve for OS in stages I-III EC patients. (**B**) Survival curve for OS in stage I EC patients. (**C**) Survival curve for OS in stage II EC patients. (**D**) Survival curve for OS in stage III EC patients. (**E**) Survival curve for OS based on age stratification in stages I-III EC patients. (**F**) Survival curve for ECSS in stages I-III EC patients. (**G**) Survival curve for ECSS in stage I EC patients. (**H**) Survival curve for ECSS in stage II EC patients. (**I**) Survival curve for ECSS in stage III EC patients. (**J**) Survival curve for ECSS based on age stratification in stages I-III EC patients. (**K**) Competitive risk of cancer-induced deaths and non-cancer-related deaths of stages I-III EC patients. (**L**) Competitive risk of cancer-induced deaths and non-cancer-related deaths of stage I EC patients. (**M**) Competitive risk of cancer-induced deaths and non-cancer-related deaths of stage II EC patients. (**N**) Competitive risk of cancer-induced deaths and non-cancer-related deaths of stage III EC patients.

Considering that age is an independent risk factor for many diseases, stratified analysis by age was conducted. The Kaplan-Meier survival curve revealed that there were significant differences in OS and ECSS between the ≥ 60 years and < 60 years groups of stages I and II and total stages I-III patients (*P* < 0.05) (Figure 7A–7D, 7F–7I). The 3- and 5-year OS rates of all patients aged under 60 years were 52.90% (95% CI: 49.50-56.40) and 45.80% (95% CI: 42.40-49.50), respectively. The 3- and 5-year OS rates of all patients aged over 60 years were 35.70% (95% CI: 34.10-37.30) and 27.70% (95% CI: 26.20-29.30), respectively. The 3- and 5-year ECSS rates of all patients aged under 60 years were 57.70% (95% CI: 54.0-61.60) and 51.60% (95% CI: 47.80-55.60), respectively. The 3- and 5-year ECSS rates of all patients aged over 60 years were 37.40% (95% CI: 35.60-39.30) and 31.70% (95% CI: 29.90-33.60), respectively.

The 4,305 patients were divided into four age groups to compare their OS and ECSS (Figure 7E, 7J). The HR in patients aged 60-69 years versus patients under 60 years old were 1.14 (OS) (*P* < 0.05) and 1.09 (ECSS) (*P* > 0.05). The HR in patients aged 70-79 years versus patients under 60 years old were 1.68 (OS) and 1.75 (ECSS) (*P* < 0.001). The HR in patients aged over 80 years versus patients under 60 years old were 2.96 (OS) and 3.40 (ECSS) (*P* < 0.001). According to the Kaplan-Meier survival curve, stages I and II and total stages I-III EC patients aged over 60 years had significantly increased risks of cancer-induced deaths and non-cancer related deaths compared to patients under 60 years old (*P* < 0.05) (Figure 7K–7N).

### Nomograms predict prognoses of stages I-III esophageal cancer patients

**Figure 8:**
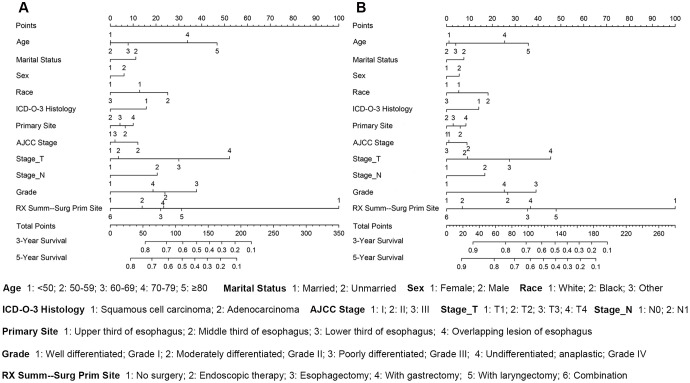
**The survival nomogram of stages I-III esophageal cancer patients.** (**A**) The sum of these numbers is located on the Total Points axis, and a line is drawn downward to the survival axes to determine the likelihood of a 3- or 5-year OS. (**B**) The sum of these numbers is located on the Total Points axis, and a line is drawn downward to the survival axes to determine the likelihood of a 3- or 5-year ECSS.

**Figure 9:**
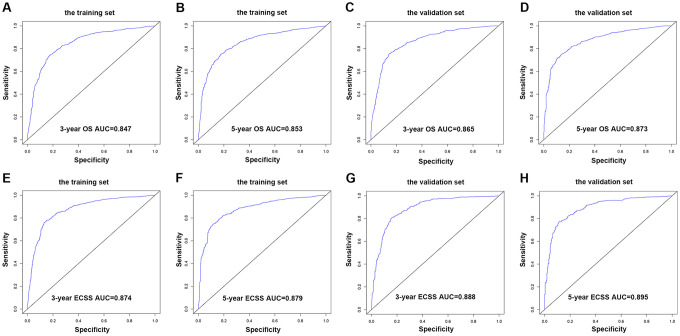
**AUC value of the ROC predicting.** (**A**) 3-year OS rates of the nomogram in the training set. (**B**) 5-year OS rates of the nomogram in the training set. (**C**) 3-year OS rates of the nomogram in the validation set. (**D**) 5-year OS rates of the nomogram in the validation set. (**E**) 3-year ECSS rates of the nomogram in the training set. (**F**) 5-year ECSS rates of the nomogram in the training set. (**G**) 3-year OS rates of the nomogram in the validation set. (**H**) 5-year OS rates of the nomogram in the validation set.

**Figure 10:**
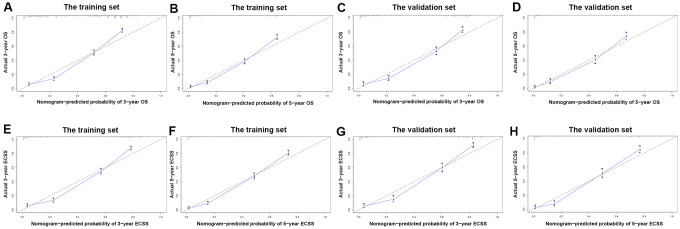
**The calibration curve for predicting patient survival.** (**A**) at 3-year OS in the training set; (**B**) at 5-year OS in the training set; (**C**) at 3-year OS in the validation set; (**D**) at 5-year ECSS in the validation set; (**E**) at 3-year ECSS in the training set; (**F**) at 5-year ECSS in the training set; (**G**) at 3-year ECSS in the validation set; (**H**) at 5-year ECSS in the validation set.

Nomograms were plotted to further evaluate the predictive significance of each variable. The multivariate analysis results were combined and all variables, except radiotherapy variables, were scored for 4,305 patients for OS (Figure 8A) and ECSS (Figure 8B). To evaluate the accuracy of the nomograms, the patients were randomly divided into the training set and validation set in a ratio of 7:3 to plot two sets of nomograms. The accuracy of nomograms was evaluated using C-index, AUC values of the ROC, and calibration curve. The predicted C-index of OS was 0.756 (95% CI: 0.75-0.76), and the AUC values of the ROC projected the 3- and 5-year OS were 0.852 and 0.859, respectively. The predicted C-index of ECSS was 0.78 (95% CI: 0.77-0.79), and the AUC values of the ROC projected the 3- and 5-year ECSS were 0.878 and 0.884, respectively. In nomograms of the training set, the predicted C-indexes of the OS and ECSS were 0.754 (95% CI: 0.74-0.76) and 0.78 (95% CI: 0.77-0.79), respectively, and the AUC values of the ROC were 0.847 and 0.853 for the projected 3- and 5-year OS, respectively, and were 0.874 and 0.879 for the projected 3- and 5-year ECSS, respectively (Figure 9A, 9B, 9E, 9F). In nomograms of the validation set, the predicted C-indexes of the OS and ECSS were 0.76 (95% CI: 0.75-0.78) and 0.79 (95% CI: 0.77-0.80), respectively, and the AUC values of the ROC were 0.865 and 0.873 for the projected 3 and 5-year OS, respectively, and were 0.888 and 0.895 for the projected 3 and 5-year ECSS, respectively (Figure 9C, 9D, 9G, 9H). The calibration curve for the probability of the 3- and 5- year OS and ECSS showed an optimal agreement between the prediction by the nomograms and the actual observations in the training (Figure 10A, 10B, 10E, 10F) and validation sets (Figure 10C, 10D, 10G, 10H).

## DISCUSSION

EC is a common malignant tumor in the digestive tract. About 70% of cases occur in men, and male patients are 2-3 times more likely to have morbidity and mortality than female patients [1]. Our dataset also mainly consisted of male patients. A 2013 study on survival outcomes of patients with EC showed that older age was significantly associated with shorter survival [9]. We confirmed these findings in the present study and found that over 70 years of age was an independent prognostic factor for OS and ECSS in stages I and II EC patients. There was less age impact in stage III patients due to other higher risk clinical conditions in these patients. Many studies have shown that marital status is significantly related to the prognosis of EC patients as married patients benefit more from a relatively harmonious family environment than divorced patients [10–12]. Our study also revealed that married patients had better survival, especially for OS in the stages I and II patients. Studies have shown that the survival rate of black patients is the worst among EC patients of different races [13, 14], which is consistent with our results. The primary site of EC makes a substantial difference in the survival rate and is a prognostic variable [15, 16]. However, our study found that only the overlapping lesion of the esophagus in stage I patients was an independent prognostic factor for poor OS and ECSS. At stage I, the prognosis of patients with SCC was better compared to patients with AC, but the differences were not observed in stages II and III patients. Therefore, early screening for EC and pathological diagnosis are very important and can guide the treatment and assessment of prognosis. The degree of differentiation is a common prognostic factor in many cancers and has a significant impact on patient survival. In our study, stages II and III patients had considerably lower OS and ECSS than stage I patients.

Surgery is the primary treatment for EC. Our data showed that the OS and ECSS of patients who had surgery were significantly longer than those who had no surgery. Most patients underwent surgery were stage I and may have better physical conditions and be younger in age, which may contribute to a better prognosis. Our results indicated that surgery was associated with age stratification, but not with basic diseases. We revealed that prognosis (cancer-induced deaths and non-cancer-related deaths) was poor in stages I and II patients older than 60 years of age. For stage III patients, age was no longer a risk factor for prognosis, perhaps because these patients had other comorbidities that could lead to worse physical conditions. The radical operation of EC involves multiple body parts, such as the neck, chest, and abdomen. The operation is complicated, time consuming, and traumatic, and has many postoperative complications. Early postoperative complications include pulmonary infection, anastomotic fistula, incision infection, and chylothorax, which have a large impact on the early recovery and prognosis of EC [7, 17–22]. In our data, patients who received esophagectomy with laryngectomy had significantly worse prognoses compared to the other surgical methods. In recent years, there has been rapid progress in endoscopic diagnosis and treatment for the early and middle stages of EC with superficial location and low lymph node metastasis rate. Endoscopic treatment and traditional esophagectomy are suitable treatments for EC, with patients experiencing less trauma and maintaining their quality of life [23–25]. Additionally, endoscopic therapy is more suitable for the elderly due to their underlying diseases and poorer physical condition.

Endoscopic therapy is an optimal treatment for patients with stage Tis (carcinoma in situ) and Tla (invasion of the mucosal lamina propria or mucosal muscle layer) EC. Patients with Tlb (submucosal invasion) stage should have esophagectomy as their first choice, but patients with Tlb who are not suitable for surgical treatment can also be treated endoscopically [26]. Although esophagectomy is the main treatment for limited resectable EC, its postoperative recurrence rate of metastasis is high, and the postoperative mortality and complication rates are much higher compared to endoscopic therapy [23, 24, 27]. Esophagectomy with gastrectomy often means that the cancer spreads locally and requires gastrointestinal reconstruction to improve the quality of postoperative life [28]. In our data, 458 patients underwent endoscopic therapy. Overall, endoscopic therapy was more effective than esophagectomy and esophagectomy with gastrectomy. However, endoscopic therapy did not significantly prolong OS of stage I patients, possibly because these patients already had good prognosis. There was no significant difference among the three surgical methods for stages II and III patients, and we considered that the physical conditions of these patients were more diverse and need to choose the best treatment based on their physical condition. We also found that the risk of non-cancer-related deaths increased significantly in stage III patients with surgery compared to no-surgery stage III patients, possibly due to poor tolerance to surgery, which could easily lead to complications or worsening of the underlying diseases.

The C-index and AUC of ROC can evaluate the accuracy of nomograms [29]. In the nomograms constructed using the OS and ECSS of stages I-III EC patients, the C-index of the 3- and 5-year OS and ECSS was higher than 0.75, and the AUC of the ROC predicted the 3- and 5- year OS and ECSS was higher than 0.85. These results indicated a good prediction accuracy. The 4,305 patients with OS and 3,326 patients with ECSS were divided into a training set (3,016 and 2,330, respectively) and a validation set (1,289 and 996, respectively) for internal verification of the model, and the nomograms showed good consistency between the training and validation sets. Moreover, both groups’ C-index and AUC showed better model discrimination, which was consistent with our nomogram results. Radiotherapy plays a crucial role in the treatment of EC before or after surgery and can achieve better local control and prolong OS. In our study, compared with no radiotherapy, single or combined radiotherapy before and after surgery did not improve the OS and ECSS of patients. The majority of our patients did not receive radiotherapy, which may lead to a significant bias in the results. These patients may have been in poor physical conditions due to severe postoperative complications and could not tolerate radiotherapy.

This retrospective report had some limitations. Firstly, we excluded patients with chemotherapy because the SEER database chemotherapy information is incomplete and ambiguous, and adverse reactions from chemotherapy may significantly increase mortality. Secondly, as a retrospective study, inherent selection bias was inevitable. Thirdly, although we performed model validation of the data, it would have been more convincing if the conclusions are supported by clinical data. At last, the role of surgery has not been proven by randomized trials. This study is based on the research of retrospective data, and the prognostic analysis of stages I-III EC patients needs to be updated with the development of medicine. The underlying cellular and molecular mechanisms also need to be studied further.

## CONCLUSIONS

This study utilized a large number of cases and incorporated various clinical information to construct and validate a universally applicable stages I-III EC prediction model that can forecast the dynamic survival rate of patients at different time points during follow-up after diagnosis. The model can guide clinicians to select treatment plans based on simulated treatment results and the risk of patient deaths during follow-up, which is beneficial to the development of personalized diagnosis and treatment plans.

## MATERIALS AND METHODS

### Case selection

The SEER database is one of the most representative large tumor registration databases in the North America. This analysis used data from the SEER 18 (1975-2016, Nov2018 Sub) database released in April 2019 with the SEER* Stat software (Version 8.3.6) [30]. Specific inclusion criteria were: (1) site and morphology. Site recoded ICD-O-3/WHO 2008: Esophagus; (2) year of diagnosis: 2004-2014; (3) international classification of diseases for oncology, 3^rd^ edition (ICD-O-3) codes 8052, 8053, 8070-8078, 8083, 8084, 8140, 8141, 8143, 8144, 8146, 8147, 8149, 8190, 8210, 8211, 8213, 8310, 8313, 8330-8333, 8480, 8481, and 8570-8574; (4) derived AJCC staging manual, 6^th^ ed: I, II, III; (5) without chemotherapy (Chemotherapy recode: No/Unknown).

The clinical data and classification used for analysis included the follows: (1) age (25-85+); (2) marital status (married and unmarried [unmarried included widowed, single, divorced, separated, unmarried, or domestic partner]); (3) sex (female and male); (4) race (white, black, and others); (5) ICD-O-3 Hist/Behav, malignant (SCC and AC); (6) primary site-labeled (upper third of the esophagus, including C15.0 and C15.3; middle third of the esophagus, including C15.1 and C15.4; lower third of the esophagus, including C15.2 and C15.5; overlapping lesion of the esophagus: C15.8); (7) Stage_T (T1, T2, T3, and T4) (according to the AJCC staging manual, 6^th^ ed); (8) Stage_N (N0, N1) (according to the AJCC staging manual, 6^th^ ed); (9) Stage_M: M0; (10) grade (well differentiated, grade I; moderately differentiated, grade II; poorly differentiated, grade III; undifferentiated, anaplastic, grade IV); (11) RX summ—surg prim site according to surgery codes (no surgery: 0; surgery: endoscopic therapy: 11-14, 20-27; esophagectomy: 30, 40, 50, 80; with gastrectomy: 52-54; with laryngectomy: 51; combination: combining 51 with any of 52-54: 55); (12) radiation sequence with surgery (no radiation; radiation after surgery; radiation before surgery; radiation before and after surgery); (13) survival months; (14) vital status recodes (alive and dead); (15) SEER cause-specific death classification; (16) COD to site recode. Several cases with unknown data were excluded.

### Statistical analysis

The Joinpoint software (Version 4.5.0) was used to analyze the annual percent changes (APCs). All data were analyzed using the software R language (Version 3.6.1) (https://www.r-project.org/). Pearson’s chi-squared tests, Fisher’s exact probability tests, Cox proportional hazards models, Kaplan-Meier survival curves and the log-rank test, nomogram, C-index, ROC curves, and calibration curves were analyzed using R projects including “rms”, “foreign”, “survival”, “survivalROC”, “caret”, “cmprsk”, and “forestplot”.

## Supplementary Material

Supplementary Figure 1

Supplementary Table 1
