# Supplementary material for: Prognostic evaluation of esophageal cancer patients with stages I-III
Source: Aging (Albany NY). 2020 Jul 23;12(14):14736–53. doi: 10.18632/aging.103532 (PMC7425498; doi:10.18632/aging.103532)
Supplement: Supplementary Figure 1 [file aging-12-103532-s002..pdf]

SUPPLEMENTARY FIGURE

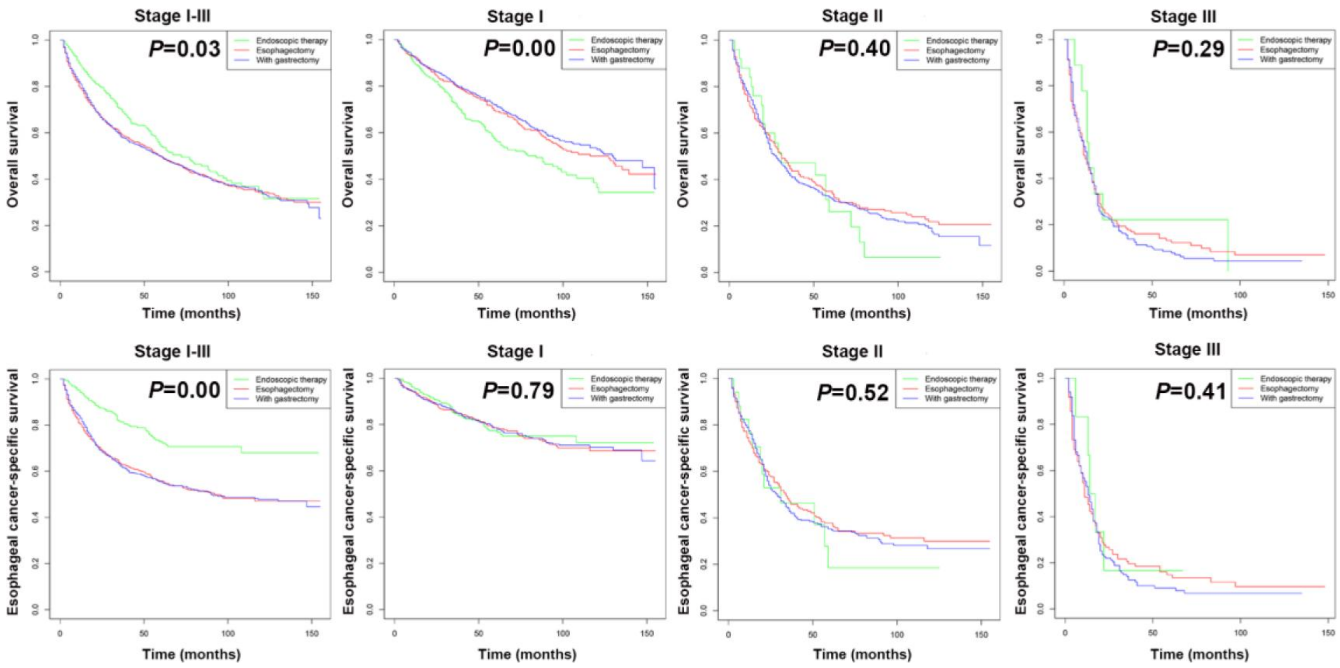

Supplementary Figure 1. Kaplan-Meier survival analyses for overall survival and esophageal cancer-specific survival in stages I-III and each stage of esophageal cancer based on three surgical methods.
