# Supplementary material for: Prognostic evaluation of esophageal cancer patients with stages I-III
Source: Aging (Albany NY). 2020 Jul 23;12(14):14736–53. doi: 10.18632/aging.103532 (PMC7425498; doi:10.18632/aging.103532)
Supplement: Supplementary Table 1 [file aging-12-103532-s001..pdf]

SUPPLEMENTARY TABLE

Supplementary Table 1. Characteristics of esophageal cancer patients with stages I-III (n=7,433).

Characteristics	No. of Patient (%)
Age (years)	
<50	282 (3.79)
50-59	1098 (14.77)
60-69	1951 (26.25)
70-79	2159 (29.05)
80≤	1943 (26.14)
Sex	
Female	1870 (25.15)
Male	5564 (74.85)
ICD-O-3 Hist/Behav, malignant	
Squamous cell carcinoma	2771 (37.28)
Adenocarcinoma	4662 (62.72)
AJCC Staging Group, 6th ed	
I	4166 (56.05)
II	1760 (23.68)
III	1507 (20.27)
